# Longitudinal trends in laboratory test utilization at a large tertiary care university hospital in Sweden

**DOI:** 10.3109/03009734.2010.528071

**Published:** 2011-02-11

**Authors:** Mirja Mindemark, Anders Larsson

**Affiliations:** Department of Medical Sciences, Section of Clinical Chemistry, Uppsala University, UppsalaSweden

**Keywords:** Clinical chemistry, diagnostic tests, health care costs, laboratory test utilization

## Abstract

**Background:**

The aim of the study was to describe and evaluate longitudinal trends in laboratory test utilization over a 7-year period from 2002 to 2008.

**Method:**

Retrospective study using test request data from the Clinical Chemistry and Pharmacology Laboratory at Akademiska Sjukhuset, a large tertiary care university hospital in Sweden. Changes in test utilization, charges, and expenditures during the study period were used as main outcome measures.

**Results:**

Laboratory test utilization increased by over 70%, with a mean annual increase of 9.3% during the study period. After adjustment for inflation, the laboratory expenditures increased by 20.2% during the study period but represented only approximately 2.0% of the hospital's total expenditure in 2008. The test menu comprised 663 tests in 2008, an increase by 146% from 2002. The mean inflation-adjusted unit price charged per test increased from €34.9 to €37.5 during the study period. The top 10, 20, and 30 tests accounted for, on average, 46.9%, 66.9%, and 75.5% of the total test volume during the study period, and 47.8%, 66.4%, and 75.7% of the total test volume in 2008. In 2008, 10 analyses, i.e. 1.5% of the number of tests on the menu, accounted for almost half the number of generated test results.

**Conclusions:**

The total number of generated test results increased by over 70% in less than a decade. Even so, the laboratory's share of the hospital's total expenditure remained low and virtually unchanged. A very small number of tests accounted for a disproportionately large share of the total number of generated test results.

## Introduction

Being the main source of objective data aiding clinicians in 60%–70% of all critical decisions such as diagnosis, treatment, and follow-up (1), laboratory tests are an essential part of an efficient health care system. As the resources of the health care sector are scarce, demands are raised to lower the costs while maintaining the quality of care. The laboratories are often among the first sections to be targeted for budget reductions, as their costs are easily discernible. However, the impact of laboratory tests on health care as a whole is wide-spread, and the monetary value of their effects is difficult to measure. Furthermore, it has been demonstrated that a reduction in test utilization produces disproportionately small true cost reductions (2), and it is by no means certain that simply reducing the number of ordered tests will lead to a decrease in the overall health care costs.

Akademiska Sjukhuset is with its 300-year history the oldest university hospital in Sweden and also one of Sweden's largest tertiary care medical centers. In 2008 the hospital had approximately 1,100 beds, with 58,000 admissions per year and more than 750,000 out-patient visits annually. Akademiska Sjukhuset serves a population of 327,000 people in the county of Uppsala, as well as the population in the surrounding counties.

As accurate and timely information on laboratory test utilization is vital for financial management of laboratories, the aim of this study was to describe longitudinal trends in test utilization from the perspective of the Clinical Chemistry and Pharmacology Laboratory at Akademiska Sjukhuset in Uppsala, Sweden.

## Materials and methods

Test utilization data from 1 January 2002 through 31 December 2008 were retrospectively extracted from the Laboratory Information System. Information on expenses, charges, admissions, and out-patient visits was collected for the years 2002 and 2008 individually. In all presentations of costs, €1 is equal to SEK10. Charges and expenditures were analyzed after adjusting for inflation according to the consumer price index (CPI), and the 2008 value is presented. The CPI, set at 100 for 1980, was 272.85 in 2002 and 300.50 in 2008.

Tests were counted as follows: All incoming test orders that generated a result, including non-chargeable results such as calculations or missing or ruined samples, were counted. Before they were counted, all test orders were broken down into individual analyses, so that the test volume presented here contained no profiles, bundled tests, or test panels.

## Results

**Figure 1.**
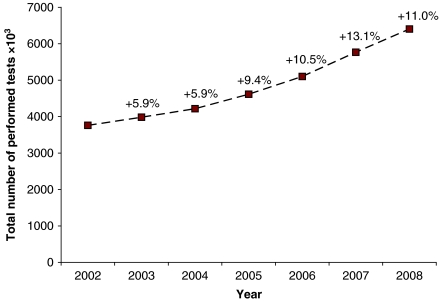
The total number of performed tests per year during the study period.

The variables considered in this study and their inflation-adjusted values for 2002 and 2008, respectively, are presented in Table I. A total of 33,846,377 test results were generated in the Clinical Chemistry and Pharmacology Laboratory during the 7-year study period. From 2002 to 2008 the number of generated test results grew by 70.3%, with an average increase of 9.30% or 440,352 test results per year (Figure 1). On average 4,835,197 tests results were generated annually during the study period. A total of 1,005 send-out tests were ordered during 2002. The corresponding figure was 3,160 in 2008. The mean price charged per test according to the price-list (not volume-adjusted) increased by 7.4%, or 2.6 inflation-adjusted euros, from €34.9 to €37.5 per test, during the study period. The total testing expense (total expense/total number of generated test results) was €2.8 in 2002 and €2.0 in 2008.

**Table I.** Investigated variables and their inflation-adjusted values presented in 2008 euros.

	2002	2008
Generated test results^a^	3,760,508	6,402,617
Analyses offered	309	663
Send-out tests ordered	1,005	3,160
Mean price charged per test^b^	€34.9	€37.5
Total laboratory expenditures	€10.4 million	€12.5 million
Total testing expense^c^	€2.8	€2.0
Admissions	53,504	58,001
Hospital beds	1,200	1,100
Out-patient visits with a physician	291,000	332,243
Additional outpatient visits	329,000	419,213
Total hospital expenditures	€513 million	€629 million

^a^Including non-chargeable test results such as calculations and missing or ruined. ^b^According to price-list, not volume-adjusted. ^c^Total expense/total number of generated test results.

The 10, 20, and 30 most commonly ordered tests accounted for, on average, 46.9%, 66.9%, and 75.5% of the total number of generated test results during the study period, and 47.8%, 66.4%, and 75.7% of the total number of generated test results in 2008 (Table II).

**Table II.** The percentage of the total number of tests represented by the 10, 20, and 30 most commonly ordered tests.

Year	Total number of ordered tests	10 most commonly ordered tests	20 most commonly ordered tests	30 most commonly ordered tests
2002	3,760,508	40.4%	59.5%	67.6%
2003	3,983,149	47.7%	69.7%	78.7%
2004	4,218,082	47.6%	69.1%	77.6%
2005	4,614,524	48.8%	69.3%	77.1%
2006	5,100,550	48.0%	67.4%	75.8%
2007	5,766,947	47.9%	66.7%	76.2%
2008	6,402,617	47.8%	66.4%	75.7%
Mean	4,835,197	46.9%	66.9%	75.5%

The number of hospital admissions increased by 8.4% from 53,504 to 58,001 between 2002 and 2008. In 2002, there were 291,000 out-patient visits with a physician. During the study period this number grew by 14.2% to 332,243. The number of additional out-patient visits increased by 27.4% from 329,000 in 2002 to 419,213 in 2008. Between 2002 and 2008, the number of hospital beds decreased from 1,200 to 1,100, and the total hospital costs increased by 22.6%. The total expenses of the Clinical Chemistry and Pharmacology Laboratory amounted to €10.4 million in 2002 and €12.5 million in 2008, representing an increase of 20.2%. The percentage of the hospital's expenditures that was constituted by the costs of the Clinical Chemistry and Pharmacology Laboratory decreased slightly by 0.1% to 2.0% during the study period.

## Discussion

The cost of health care in Sweden, as well as in many other countries, increases every year (3–5) and more rapidly so than the Gross Domestic Product (GDP) (6). In 2002, the net costs of health care in Sweden amounted to €15,459 million (2008 euros) (7). By 2008, the total expenditure had reached €20,640 million (7), an increase of 34% in less than a decade. The GDP, on the other hand, had during the same period only increased by approximately 17% (8). The total costs of health care in Sweden, expressed as a percentage of GDP, were in 2005 9.2% (9), i.e. slightly above the 8.9% average of the OECD countries (10), but substantially lower than, for example, the corresponding 15.2% of GDP spent on health care in the US (10). The annual total cost of laboratory testing in Sweden is approximated at €0.8 billion (in 2008 euros), about half of which is represented by the costs of clinical chemistry tests (11). Of the total health care costs, laboratory expenditures only account for approximately 4% in Sweden (12). The corresponding figures are 20% in the United States, 4% in the United Kingdom, 5.2% in Australia, and 7%–10% in Canada (13). Though laboratory tests only account for a very small part of the total health care costs, the laboratories are often among the first to be targeted for budget reductions. However, the impact of laboratory tests on health care as a whole is wide-spread, and the monetary value of their effects is difficult to measure. Thus, the relation between test utilization, costs, and quality of care is as complex as it is central to health care management.

Very little has so far been published about changes in test utilization over time. Accurate and timely information on trends in test utilization is essential to financial management of clinical laboratories. The aim of this study was therefore to elucidate the changes over time in clinical chemistry and pharmacology test utilization, data that are not readily available. Studies such as this provide base-line data, a necessity when planning for future adaptation and improvements of test utilization and laboratory structure. To our knowledge, this is the first Swedish study of clinical chemistry and pharmacology test utilization, charges, and expenses. This 7-year analysis was designed to describe and evaluate trends in test utilization, expenditures, and charges at a clinical chemistry laboratory at a large tertiary care university medical center. The study provides insight into the utilization and economics of laboratory testing during the past 7 years, a period that was characterized by tightened budget control and ever-growing concern about medical costs.

The principal findings of this study were substantial increases in the number of generated test results and in the number of tests offered, despite a virtually unchanged share of the hospitals total expenses represented by the costs of laboratory testing. From 2002 to 2008 the work-load, as defined by the number of generated test results, increased by over 70%, with a mean annual increase of 9.30%. This is higher than the average laboratory test growth rate of 7.2% seen in the Netherlands during the 1980s (14) but lower than the average increase rate of 12.1% in the US in 1993 (15). Other similar studies have presented laboratory test growth rates of 2.3%–13.8% at different times during the period 1970–2005 (15–18), whereas yet others have demonstrated virtually unchanged (19) or declining utilization rates (20), the latter most likely due to interventions aimed at reducing test ordering. The percentage of the total number of generated test results that was represented by send-out tests increased slightly from 0.03% to 0.05% and thus only accounted for a very small portion of the total number of generated test results throughout the study period.

As the mean price charged per test is skewed upwards by a relatively small number of high-cost analyses, it is not necessarily representative of the general level of unit prices charged, but it is nevertheless suitable for a comparison of trends. The mean price charged per test had increased slightly during the study period but at a rate lower than would be expected considering the inflation rate as defined by the CPI. However, the data available in this study do not allow analysis of the underlying cause of this. As opposed to the mean price charged per test, the total testing expense takes into account the prices charged per test as well as the test utilization volumes. This variable had, unlike the mean price charged per test, decreased from €2.8 to €2.0 during the study period, most likely due to a combination of increased automation and efficiency.

The number of different analyses offered by the Clinical Chemistry and Pharmacology Laboratory was 663 in 2008, and had thus more than doubled during the study period. Despite this, the share of the total number of generated test results that was represented by the 10 most commonly ordered analyses was very stable at approximately 50% throughout the study period, indicating that most of the new tests that were added to the test menu were low-frequency tests.

The 10, 20, and 30 most commonly ordered tests accounted for, on average, 46.9%, 66.9%, and 75.5% of the total test volume during the study period, and 47.8%, 66.4%, and 75.7% of the total test volume in 2008. In a study by Nexø, the top-20 tests represented more than 80% of all test requests (19), whereas, in a South African study, the 10 and 30 most commonly ordered tests represented 36.3% and 67.8%, respectively, of all ordered tests (17), results similar to those presented in this study. Among the 10 most commonly ordered tests in this study, the intergroup order of tests varied minimally during the study period and mainly consisted of the components of the full blood count with the addition of potassium, and occasionally C-reactive protein (CRP). Among the 11–20 most commonly ordered tests, the extent of intergroup variation was slightly greater than in the top-10 group and increasingly so further to the bottom of the group. The group containing the top 21–30 tests, however, was much less homogeneous than the two previous groups. The continuity of different analyses was still fairly high among the top 21–30 tests, but no test was consequently represented in this group throughout the study period. The data on the top-30 tests could be useful as an indication of where small changes in test utilization may bring about considerable savings, as small technologies are likely to account for far more of the over-utilization than big expensive technologies (21), and low-cost high-frequency tests have been demonstrated to account for the major proportion of laboratory costs (22).

The number of admissions and out-patient visits had increased by 20.2% during the study period. Assuming the test-ordering pattern to be the same for the admissions and out-patient visits in 2002 and 2008, the increase in the number of admissions and out-patient visits would merely explain about one-third of the increase in test volume. The major part of the increase in work-load could thus most likely be ascribed to intrinsic growth.

There are a few potential limitations of this study. Firstly, the test utilization pattern at Akademiska Sjukhuset may not be representative of that in Sweden as a whole. Despite this limitation, the results warrant evaluation and are indicative of trends in clinical chemistry and pharmacology test utilization in Sweden. Secondly, the way the tests are counted will admittedly inflate test count, but this should not influence the evaluation of trends in utilization, with which this study is concerned. Thus, as filtering of test-ordering data would risk biasing the results, we chose to present the raw, gross-type test-ordering data. Furthermore, presentation of test volumes on the basis of individual tests as opposed to test volumes containing panels as bundled tests should facilitate comparisons between laboratories and countries as test panels include different tests in different settings. Thirdly, as the total number of generated test results included some non-chargeable results such as calculations, the total testing expense may be slightly under-estimated. However, there is no reason to believe that the number of non-chargeable results varied significantly during the study period, and this would thus not affect the evaluation of trends. Lastly, it should be noted, for this type of study in general, that raw numbers of ordered tests do not reflect the quality of the care provided and that they say nothing of whether the tests were appropriately used.

The main strength of this study is that the data on the utilization of a substantial number of clinical chemistry and pharmacology tests, representing a large patient population, were evaluated over an extended period of time. The study defines trends and may thus have potential predictive values.

## Conclusion

In conclusion, the total number of generated test results increased by over 70% in less than a decade, whereas a very small number of tests accounted for a disproportionately large share of the total number of performed tests. Despite the substantial increase in the number of generated test results, the laboratory's share of the hospital's total expenditure remained low and virtually unchanged at approximately 2% during the study period.
